# TRPV1 Channels Are Functionally Coupled with BK(mSlo1) Channels in Rat Dorsal Root Ganglion (DRG) Neurons

**DOI:** 10.1371/journal.pone.0078203

**Published:** 2013-10-16

**Authors:** Ying Wu, Yongfeng Liu, Panpan Hou, Zonghe Yan, Wenjuan Kong, Beiying Liu, Xia Li, Jing Yao, Yuexuan Zhang, Feng Qin, Jiuping Ding

**Affiliations:** 1 Key Laboratory of Molecular Biophysics of the Ministry of Education, College of Life Science and Technology, Huazhong University of Science and Technology, Wuhan, Hubei, China; 2 Department of Physiology and Biophysical Sciences, State University of New York, Buffalo, New York, United States of America; 3 School of Public Health, Xinxiang Medical University, Xinxiang, Henan, China; Indiana University School of Medicine, United States of America

## Abstract

The transient receptor potential vanilloid receptor 1 (TRPV1) channel is a nonselective cation channel activated by a variety of exogenous and endogenous physical and chemical stimuli, such as temperature (≥42 °C), capsaicin, a pungent compound in hot chili peppers, and allyl isothiocyanate. Large-conductance calcium- and voltage-activated potassium (BK) channels regulate the electric activities and neurotransmitter releases in excitable cells, responding to changes in membrane potentials and elevation of cytosolic calcium ions (Ca^2+^). However, it is unknown whether the TRPV1 channels are coupled with the BK channels. Using patch-clamp recording combined with an infrared laser device, we found that BK channels could be activated at 0 mV by a Ca^2+^ influx through TRPV1 channels not the intracellular calcium stores in submilliseconds. The local calcium concentration around BK is estimated over 10 μM. The crosstalk could be affected by 10 mM BAPTA, whereas 5 mM EGTA was ineffectual. Fluorescence and co-immunoprecipitation experiments also showed that BK and TRPV1 were able to form a TRPV1-BK complex. Furthermore, we demonstrated that the TRPV1-BK coupling also occurs in dosal root ganglion (DRG) cells, which plays a critical physiological role in regulating the “pain” signal transduction pathway in the peripheral nervous system.

## Introduction

The nonselective cation TRPV1 channel responds to many stimuli such as capsaicin (pungent ingredient of hot peppers), acids and heat [1,2]. Capsaicin can stimulate chemosensitive and thermosensitive nociceptors and elicits pain via a subset of primary afferent neurons [3], and in DRG neurons, TRPV1 channel plays an essential role in pain signal generation and regulation [4-8]. Therefore, TRPV1 channel can detect and regulate body temperature by providing a sensation of scalding heat and pain. TRPV1 receptors exist mainly in the nociceptive neurons of the peripheral nervous system and other tissues. Larger-conductance calcium-activated K^+^ (BK) channel is composed of four mSlo1 α subunits, responding to both membrane depolarization and submembrane Ca^2+^ changes in cells [9,10]. Considering that both BK (Ca^2+^ sensor) and TRPV1 (Ca^2+^ source) coexist in DRG neurons [11,12], it is tempting to ask whether two channels are functionally coupled.

Recent studies suggest that BK channels may indeed cross-talk to other members of the TRP family. For example, 11,12 epoxyeicosatrienoic acid (11,12 EET) could activate TRPV4 channels and increase the frequency of Ca^2+^ sparks via ryanodine receptors located on the sarcoplasmic reticulum. EET-induced Ca^2+^ sparks activated nearby BK channels and induced the membrane hyperpolarization to depress stimuli in vascular smooth muscle cells (VSMCs) [13]. Another study reported that the BK channel was physically associated with TRPC1 in VSMCs. Moreover, they found that Ca^2+^ influx through TRPC1 could activate BK to induce membrane hyperpolarization. The hyperpolarization effect of BK-TRPC1 coupling could serve to prevent excessive contraction of VSMCs [14]. Afterwards, BK currents enhance the hyperpolarization to reduce stimuli in VSMCs.

In this study we investigated possible coupling between TRPV1 and BK channels in both HEK293 and DRG cells. Using the patch-clamp recording, fast temperature stimulation of TRPV1 and coimmunoprecipitation techniques, we demonstrated that TRPV1 channels were associated with BK channels to form a signaling complex and that the Ca^2+^ influx through TRPV1 sufficed to activate BK channels in DRG cells. The augment of BK currents activated by calcium influx via TRPV1 could cause hyperpolarization consequently decreasing membrane potentials and therefore counteracting the depolarization effects from TRPV1 opening. The study provides new insights into the complicated pain signal transduction pathways, and suggests that individual signaling molecules may form functional complexes to further extend their functions in neurons.

## Materials and Methods

### Ethics statement

All animal procedures were approved by Hubei Research Center of Experimental Animals (Permit Number: SCXK 20080005) and in accordance with the guidelines of International Association for the Study of Pain [15]. Animals were housed in cages with free access to food and water and were kept under natural diurnal cycle. Male adult Wistar rats (4 weeks, ^~^60 g) were deeply anesthetized with intraperitoneal injection of 10% chloral hydrate and sacrificed by cervical dislocation to minimize the animal suffering. After being positioned supine on a horizontal holder, the abdomen of each animal was retracted and spine was separated from the intraperitoneal. Then dorsal root ganglia (DRG) were isolated from the spine for the electrophysiology experiments.

### Materials

All the restriction enzymes were available from NEB. The vector pcDNA3.1/Zeo(+), lipofectamine 2000 and dynabeads protein G was purchased from Life Technologies, Invitrogen (Carlsbad, CA, USA). Dulbecco’s modified Eagle’s medium (DMEM), fetal bovine serum (FBS) and DMEM-F12 were purchased from Gibco BRL (Gaithersburg, MD, USA). The monoclonal anti-myc tag antibody was from Abcam (Cambridge, UK). Monoclonal anti-HA and goat-anti-mouse HRP-conjugated IgG were from EMD Millipore (Billerica, MA, USA). rhodamine-conjugated goat-anti-mouse secondary antibodies was purchased from Proteintech Group (Chicago, IL, USA). The monoclonal anti-myc tag and anti-Slo1 antibody were purchased from Abcam (Cambridge, UK); the polyclonal anti-TRPV1 antibody from Santa Cruz (Dallas, Texas, USA); Monoclonal anti-HA, HRP-conjugated goat-anti-mouse and rabbit-anti-goat IgG from EMD Millipore (Billerica, MA, USA); Rhodamine-conjugated goat-anti-mouse secondary antibodies from Proteintech Group (Chicago, IL, USA). The enhanced chemiluminescence lightening (ECL) was obtained from Thermo Scientific (Bremen, Germany). All chemicals were obtained from Sigma-Aldrich (St. Louis, MO, USA). Coverslips were purchased from Electron Microscopy Sciences (Hatfield, PA, USA).

### Plasmid construction

Full length cDNA for mSlo1 (Accession NO. NM_001253365) and rat TRPV1 (Accession NO. NM_031982), were subcloned into pcDNA3.1/Zeo(+), respectively. To generate the mSlo1-TagGFP construction, a 700bp fragment of the C-terminal of mSlo1 which contained a unique XhoI site was PCR amplified and fused to the cDNA of TagGFP, the overlapping fragment was then subcloned into pcDNA3.1-mSlo1 using XhoI/XbaI sites. Myc-mSlo1 was generated by tagging a c-myc epitope (EQKLISEEDL) to the N-terminal of mSlo1 with KpnI/NotI. Using the sequential overlap extension PCR method, the TRPV1 subunit was tagged with a c-myc epitope at the extracellular loop and a hemagglutinin epitope (YPYDVPDYA) at the C-terminus, then cloned into KpnI/EcoRI to generate TRPV1-myc and TRPV1-HA, respectively. All constructions were verified by sequencing.

### Preparation of DRG cells

Dorsal root ganglia (DRG) were isolated from the spine of male adult Wistar rats in ice-cold DMEM (low glucose) and treated with trypsin (1 mg*ml^−1^) and collagenase (1 mg*ml^−1^) for 20 min at 37 °C. Dissociated cells were plated on poly-lysine-coated coverslips and maintained in MEM–F12 (Gibco) supplemented with 10% FBS. Experiments were carried out within 24 hours after preparation. Only small neurons were used (15–25 μm) [16].

### HEK293 cells culture and transfection

HEK293 cells were cultured in DMEM supplemented with 10% FBS and grown in a 37 °C incubator with 5% CO_2_. One day before transfection, cells were transferred to 24-well plates. When 90% confluent, cells were transiently transected using lipofectamine2000. Following experiments were performed 1-2 day after transfection.

### Electrophysiology

Electrophysiology experiments on HEK293 and DRG cells were performed using an EPC-9 patch-clamp amplifier with its software (HEKA, Germany). Pipette resistance was typically 2-4 MΩ. For whole-cell patch configuration, extracellular solution contained the following (in mM): 145 NaCl, 5 KCl, 1 MgCl_2_, 0.3 or 2 CaCl_2_, 10 HEPES((PH 7.4). High resistances seals were formed in a bath solution of ND-96. Intracellular solutions contained the following (in mM): 145 KCl, 5 NaCl, 2.5 MgCl_2_, 10 HEPES, 0.1 EGTA or 5 EGTA or 10 BAPTA, 2 K_2_ATP(PH 7.4). The series resistances were 85% compensated. For inside-out patch configuration, the pipette solution contained the following (in mM): 160 MeSO_3_K, 2 MgCl_2_, 10 HEPES (PH 7.0). Puff solution contained the following (in mM): 160 MeSO_3_K, 10 HEPES, 5 HEDTA (for 4 and 10 μM Ca^2+^) or 5 EGTA (for 1 μM Ca^2+^) with added Ca^2+^ to get 1, 4, 10 and 300 μM free Ca^2+^, as defined by the EGTAETC program (McCleskey, Vollum Institute, Portland, OR), with the pH adjusted to 7.0. During recording, different Ca^2+^ concentration solutions were applied onto membrane patches via a perfusion pipette containing eight solution channels. Currents were recorded at room temperature (22–25 °C) except thermal experiments.

### Immunofluorescence imaging

HEK293 cells were co-transfected with mSlo1-TagGFP and TRPV1-myc using a 1:1 ratio. Then cells on coverslip-bottom dishes were maintained for the following day and then, fixed with 2% paraformaldehyde in PBS for 15 minutes. After blocking for 1 hr with 5% bovine serum, cells were incubated with monoclonal anti-myc tag antibody (1:300) for 5 hrs, washed six times, and incubated with rhodamine-conjugated anti-mouse IgG antibody (1:300) for 2 hr. All the experiments were performed at room temperature (^~^25 °C). 

High-resolution fluorescence images were acquired with an Andor Revolution XD laser confocal microscope system based on a spinning-disk confocal scanning head, CSU-X1 (Yokogawa Electric, Musashino-shi, Tokyo, Japan) by using a 100× oil objective lens (numerical aperture=1.30, Zeiss, Germany). Parameter selection, sample scanning and image acquisition were all controlled by the Andor iQ application software. Images were viewed, processed and analyzed in Image J (National Institutes of Health, Bethesda, MD, USA) and Autoquant X^2^ (Media Cybernetics, Rockville, MD, USA).

### Co-immunoprecipitation and immunoblots

Myc-mSlo1 and TRPV1-HA co-transfected HEK293 cells or DRGs from male adult Wistar rats were harvested and lysed in 400 μl detergent extracted buffer (50 mM Tris-HCl pH7.4, 50 mM NaCl, 1% Nonidet P-40, 500 μM PMSF, with addition of complete mini EDTA-free protease inhibitor cocktail tablets) for 30 minutes on ice. Half of the sample was used for Input, the other half for Co-IP. Insoluble material was removed by centrifugation (14,000 rpm) at 4 °C for 20 minutes. Supernatants were collected and pre-cleared with dynabeads protein G. Extracted proteins were incubated with 7 μg of anti-HA tag, anti-myc tag, anti-Slo1 or anti-TRPV1 prebound dynabeads at 4 °C for 2 hours with gentle agitation on a platform shaker, followed by extensive washing. Immunoprecipitates were prepared in 50 μl of 5x Laemmli’s buffer and subsequently resolved on an 8% SDS-PAGE gel. Not all the 200 μL was loaded for Input (5 μL for HEK293 cells and 20 μL for DRG cells).

For immunoblots, immunoprecipitates were transferred to nitrocellulose membrane and incubated at 4 °C overnight with the primary anti-myc tag (1:1000), anti-HA tag (1:1000), anti-TRPV1 (1:1000) or anti-Slo1 (1:1000) antibody overnight. Immunoreactivity was visualized by using the ECL detection system after incubation the horseradish peroxidase-conjugated secongary antibody for 2h. Immunoprecipitation with rat or goat normal serum (IgG-IP) was used as negative controls.

### Data analysis

Electrophysiology data were analyzed with Clampfit (Axon Instruments, Foster city, CA) and SigmaPlot (SPSS Science, Chicago, IL, USA). All the data are presented as mean ± S.D.. T-test was used in the statistical analysis to confirm the significant difference.

## Results

### The TRPV1 channel functionally coupled with the BK (mSlo1) channel in HEK293 cells

**Figure 1:**
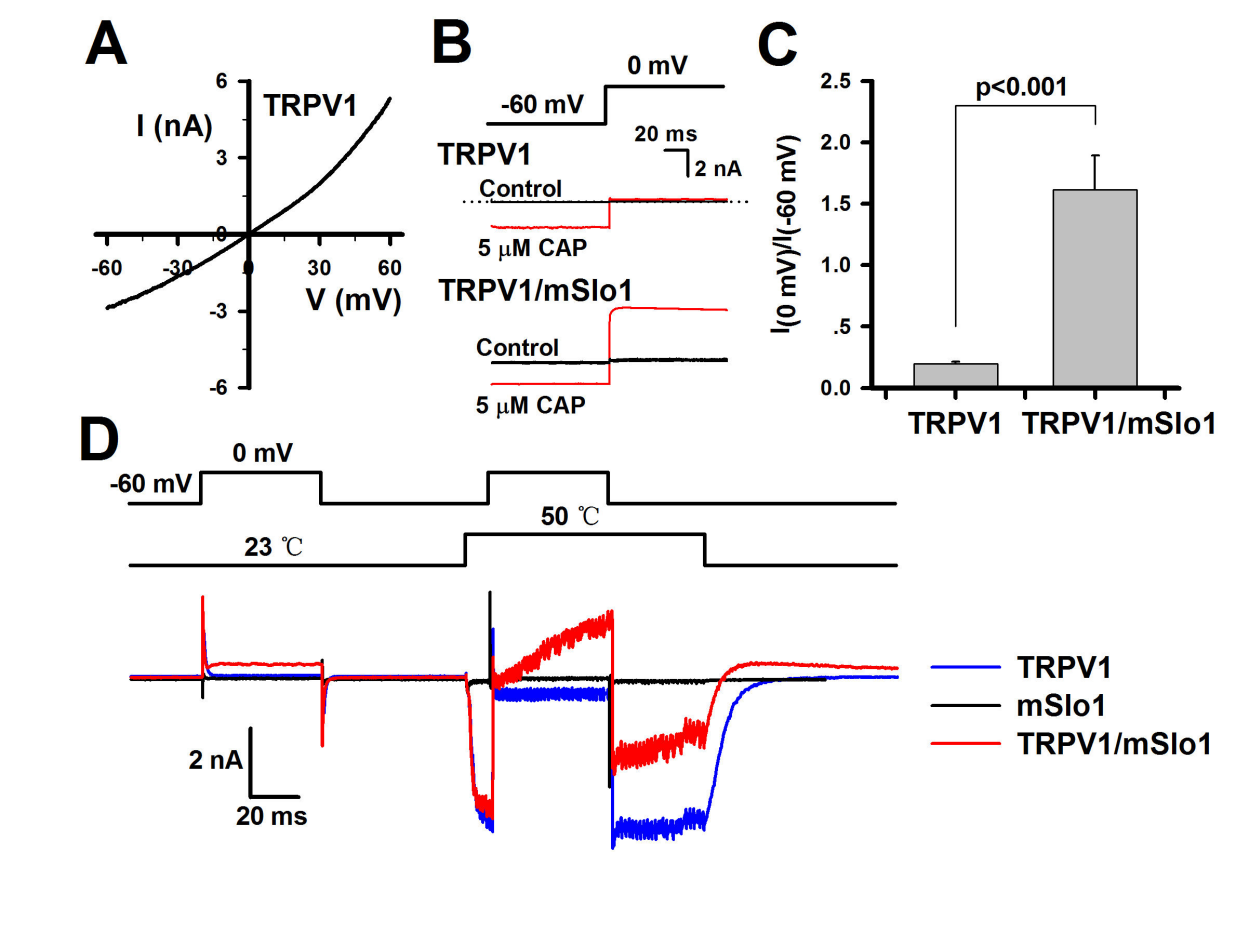
The BK(mSlo1) currents activated by Ca^2+^ influx through TRPV1 channels. A, the voltage-current (I-V) currve of TRPV1 channels, expressed in HEK293 cells, was acquired in the bath solution with 300 μM Ca^2+^, in the whole-cell patch configuration by a 240 ms voltage ramp from -60 to 60 mV, in the presence of 5 μM Capsaicin.(n≥8) B, the representative traces of TRPV1 (upper) and TRPV1/mSlo1 (lower) expressed separately in HEK293 cells were recorded at -60 and 0 mV, in the presence of 0 and 5 μM Capsaicin, respectively. The voltage protocol is placed at the top. (n≥8) C, the relative maximal current of I_max_(0 mV)/|I_max_(-60 mV)| at 5 μM Capsaicin is plotted for the TRPV1 and TRPV1/mSlo1 as indicated. (n≥8) D, the representative traces of TRPV1 (blue) and TRPV1/mSlo1 (red) expressed separately in HEK293 cells were recorded at -60/0 mV and 23/50 °C as indicated. Both of the voltage and temperature protocols are placed at the top. (n=3).

TRPV1 is a nonselective cation channel permeating Na^+^, K^+^ and Ca^2+^, of which the reversal potential is about 0 mV in 145 mM [Na^+^]_o_/145 mM [K^+^]_i_ solutions. A current-voltage (I-V) curve of TRPV1 from a voltage ramp confers a reversal potential of 0 mV as we predicted (Figure 1A). To minimize contamination by TRPV1 currents, this reversal potential of TRPV1 was thus chosen as a test potential to determine whether BK currents could be evoked by Ca^2+^ flux through TRPV1 in most of our experiments. Noticeably, 300 μM Ca^2+^ was added into the extracellular bath solution in order to reduce desensitization of TRPV1 currents in most of experiments. Currents in TRPV1-transfected HEK293 cells occurred only at -60 mV, in the presence of 5 μM Capsaicin (Figure 1B top). In contrast, in the TRPV1/mSlo1 co-transfected HEK293 cells currents occurred at both -60 and 0 mV, in the presence of 5 μM capsaicin (Figure 1B bottom). Because the reversal potential of BK channels is near -60 mV in normal saline, the currents at -60 mV are only from TRPV1 and currents at 0 mV are mainly attributed to BK. In Figure 1C, a summary confers that the relative maximal currents I_max_(0 mV)/|I_max_(-60 mV)| are 0.20 ± 0.02 (n=5) for TRPV1 and 1.62 ± 0.28 (n=8) for TRPV1/mSlo1, in the presence of 5 μM Capsaicin (p<0.05), suggesting that the augmented currents are BK currents acitvated by Ca^2+^ influx via TRPV1 channels. This result indicates that the TRPV1 channel couples with BK(mSlo1) channel functionally. Since TRPV1 is one of thermal TRP channels, an infrared laser device was thus used as a heat source to bright light and activate TRPV1 channels by a temperature step from 22 °C to >60 °C in submillisecond [17]. Combining the patch-clamp setup with the infrared laser device, we intended to examine whether BK currents could be activated by the Ca^2+^ influx through TRPV1 in submilliseconds by increasing temperature. In Figure 1D, a TRPV1-transfected cell shows a huge TRPV1 current (blue) at -60 mV evoked by a temperature jump from 23 to 50 °C for 10 ms, but no current (blue) was recorded after a voltage step to 0 mV at 50 °C; a TRPV1/mSlo1-transfected cell shows a rapidly increasing current (red) at 0 mV, following the huge TRPV1 current (red) at -60 mV by a 27 °C jump for 10 ms. This indicates that mSlo1 can be activated by the TRPV1 in submilliseconds, even in the 300 μM Ca^2+^ extracellular saline. Afterwards, the inward net currents of mSlo1 and TRPV1 at 50 °C were significantly decreased at -60 mV partly due to the activated outward currents of mSlo1.

**Figure 2:**
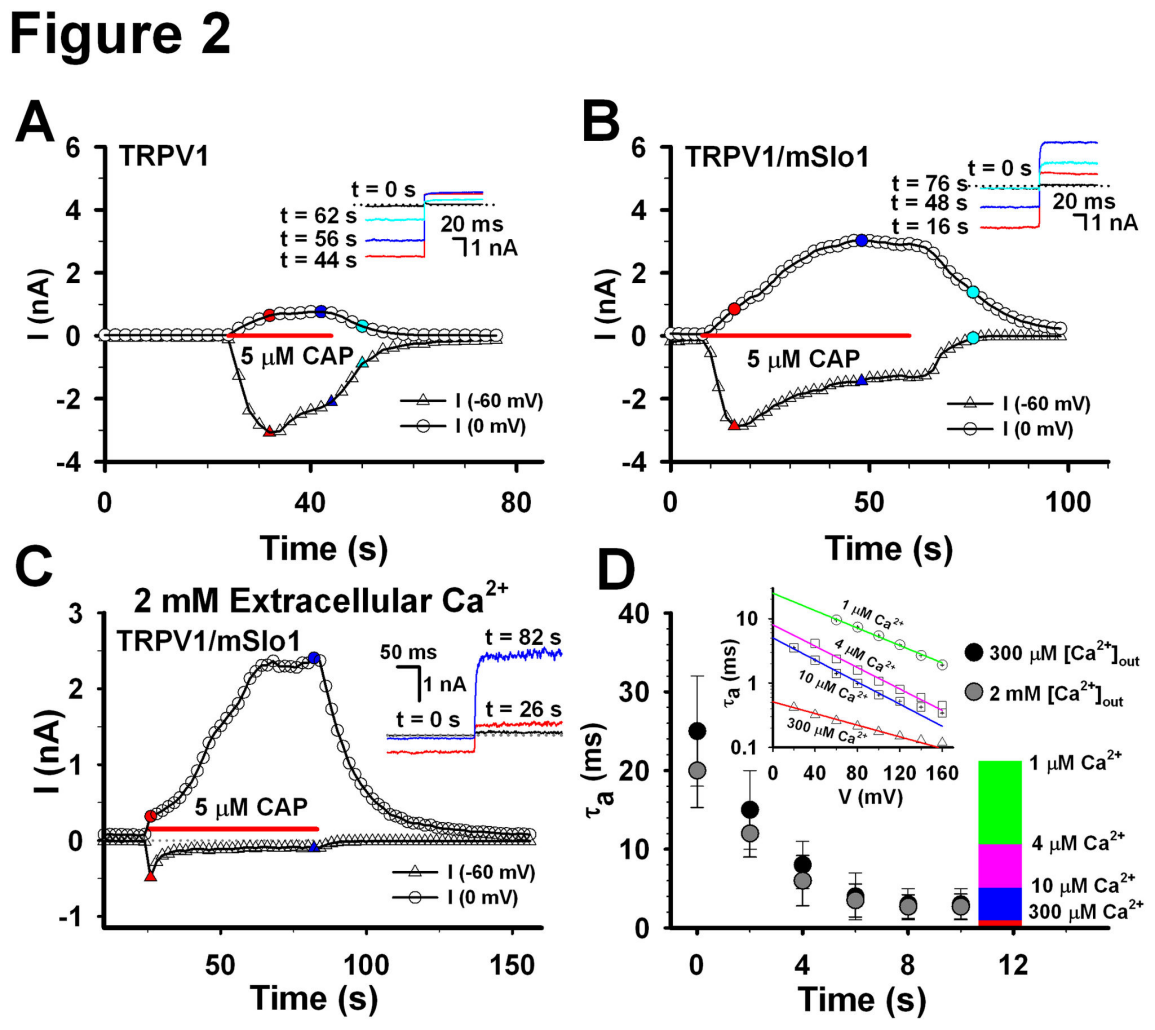
Comparison of TRPV1 and TRPV1/mSlo1 currents. A, The time coruse of the TRPV1 currents. The TRPV1 currents, expressed in HEK293 cells, were recorded with repetitive voltage steps from -60 to 0 mV with a time interval of 2 seconds, before, during, and after the application of 5 μM Capsaicin as indicated by the horizontal bar. The peak currents at -60 and 0 mV are plotted as a function of elapsed time as indicated. The inset shows the traces of currents at different time as indicated. B, The same as described in A, except for the currents of TRPV1/mSlo1. C, Time course of TRPV1/mSlo1 currents in the 2 mM Ca^2+^ extracellular solution. The same as described in B, but in the extracellular 2 mM Ca^2+^ solution. D, Estimation of local Ca^2+^ concentrations of BK channels. Left, activation time constants of BK(mSlo1) channels were plotted for 1, 4, 10 and 300 μM Ca^2+^, respectively (Wu et al., 2009). Right, activation time constants of mSlo1 channels induced by calcium influx through TRPV1 were plotted as the function of recording time. Right scale indicates the corresponding relationship between the activation time constants and local Ca^2+^ comcentrations of BK channels. (n=5-8).

The time course of TRPV1 currents indicated that TRPV1 showed a little desensitization at -60 mV, even in the extracellular 300 μM Ca^2+^, and a small amount of currents appeared at 0 mV, in the presence of 5 μM Capsaicin (Figure 2A), suggesting that the reversal potential of TRPV1 is somewhat off 0 mV. Moreover, we noticed an ohmic leap of TRPV1 current by a voltage step from -60 to 0 mV in the inset of Figure 2A. Correspondingly, the time courses of TRPV1/mSlo1 currents indicated that there was a larger amount of currents including both the BK and TRPV1 currents at 0 mV, in the presence of 5 μM Capsaicin (Figure 2B). In contrast, there was no current at either -60 or 0 mV, in the absence of 5 μM Capsaicin, indicating that BK cannot be activated without TRPV1 channels expressed in HEK293 cells. Based on the activation time constants of currents shown in the lower inset, we found that TRPV1 currents (16 s, red) occurred at the earlier stage and BK currents (48 s, blue and 75 s, cyan) at the later stage. Moreover, the currents at 0 mV gradually increased to a saturation level in the presence of 5 μM capsaicin. This may imply that the local calcium concentration around BK channels accumulates to a saturation level. Similarly, while raising the extracellular Ca^2+^ from 0.3 to 2 mM, we found that the time course of the robust BK/TRPV1 currents rose more rapidly, even though the TRPV1 currents at -60 mV quickly desensitized to ~200 pA (Figure 2C). This may imply that a calcium accumulation induces an augment of local calcium concentration of the BK channels. Considering that the open probability of BK(mSlo1) is about 10% at 0 mV, in the presence of 10 μM Ca^2+^ [18,19], we thus predicted that the local calcium concentration around BK(mSlo1) channel should be around 10 μM. Based on our previous data [19], the activation time constants of BK(mSlo1) currents depend on both the calcium concentrations and voltages (Figure 2D inset). Thus, the local Ca^2+^ concentration of BK can be determined based on its activation time constants at 0 mV [20]. Measuring the activation time constants from the TRPV1-BK coupling experiments (Figure 2B), we found that local calcium concentration of BK gradually increased to over 10 μM with time (Figure 2D), similar to the Ca^2+^-loading time-dependence of Cav1.2 coupling with BK channels [21]. All the evidences indicate that BK does couple with TRPV1 to form a TRPV1-BK complex [20]. 

### TRPV1 channel is physically associated with BK channel

**Figure 3:**
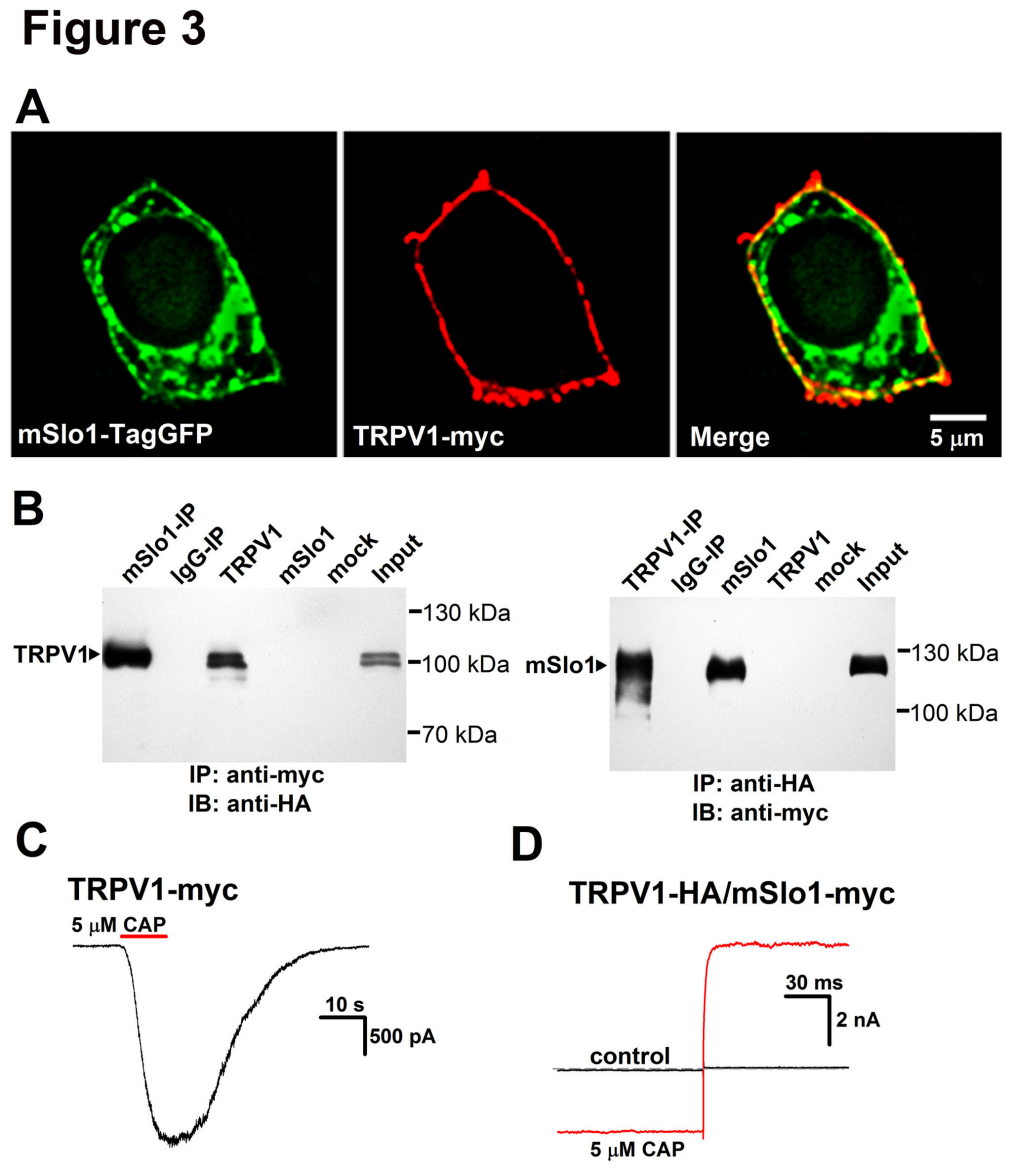
Colocalization of mSlo1 and TRPV1 in HEK293 cells. A, Surface expression of mSlo1 and TRPV1. HEK293 cells were cotransfected with mSlo1-TagGFP (green) and TRPV1-myc (red). Confocal images of mSlo1-TagGFP (left panel) and TRPV1-myc (middle panel) have clearly green and red edges, respectively, and the merged image (right panel) shows a yellow edge, indicating that two proteins were colocalized. 132 colocalized cells from 3 experiments were imaged and analyzed. Scale bar is 5 μm. B, coimmunoprecipitation experiments carried out on lysates of TRPV1 and mSlo1 co-expressed HEK293 cells. Left: TRPV1 channels can be detected in immunoprecipitates prepared by anti-myc antibody (mSlo1-IP) (n = 4). Right: mSlo1 channels can be detected in immunoprecipitates prepared by anti-HA antibody (TRPV1-IP) (n = 5). The lane marked “input” refers to a sample of the original cell lysate from expressed HEK293 cells. The lane “IgG-IP” respresents a negative control sample, which was immunoprecipitated with rat serum not expressing epitope-tagged proteins. The lane “mSlo1”, “TRPV1” and “mock”respresent samples of transfected alone or no transfected HEK293 cells. Note that no signal was detected from IgG-IP or mock samples. C, The currents of TRPV1 were not affected by myc. The representative trace of TRPV1-myc was obtained at -60 mV, in the presence of 5 μM Capsaicin. D, The currents of TRPV1/mSlo1 were not affected by HA. The representative traces of TRPV1-HA/ Slo1-myc were obtained by a voltage step from -60 to 0 mV, in the absence and presence of 5 μM Capsaicin. (n=4-8).

The TRPV1-BK coupling was further probed by both the surface expressions and coimmunoprecipitation trials with HEK293 cells. Immunofluorescence experiments were performed to determine subcellular localization of TRPV1 and BK. A HEK293 cell was co-transfected with mSlo1-TagGFP (Figure 3A Left) and TRPV1-myc (Figure 3A Middle). Merged image showed a clear overlapping of TRPV1 and BK (yellow) (Figure 3A Right), indicating that both TRPV1 and BK were mostly localized on the plasma membrane.

Next, we used coimmunoprecipitation method to determine whether mSlo1 and TRPV1 are physically associated in co-transfected HEK293 cells. The C-terminal of TRPV1 was labeled with a HA tag and mSlo1 was tagged with a myc tag at the N-terminal. Consistent with previous studies, our immunoblot experiment revealed that TRPV1 bands with or without glycosylation were located at ^≈^ 110 kDa and at ^≈^ 100 kDa, respectively [22,23], for either transfected alone or co-transfected with mSlo1 in HEK293 cells (Figure 3B, left). Immunoblotting the mSlo1-IP sample with anti-HA antibody demonstrated a strong immunoreactive band of TRPV1 subunit (^≈^ 110 kDa) (Figure 3B, left), suggesting that mSlo1 was able to pull down TRPV1 from the co-transfected lysates of TRPV1 and mSlo1. Furthermore, anti-HA antibody was able to reciprocally pull down mSlo1 (Figure 3B, right). In control IgG-IP experiments, no band was dectected.

Here both the myc and HA tags did not affect the currents of TRPV1 and mSlo1 channels in the above experiments (Figure 3C-D). Therefore, both the coimmunoprecipitation and fluorescence results implied that TRPV1 physically associated with BK.

### Localization of BK and TRPV1 within Ca^2+^ nanodomains

**Figure 4:**
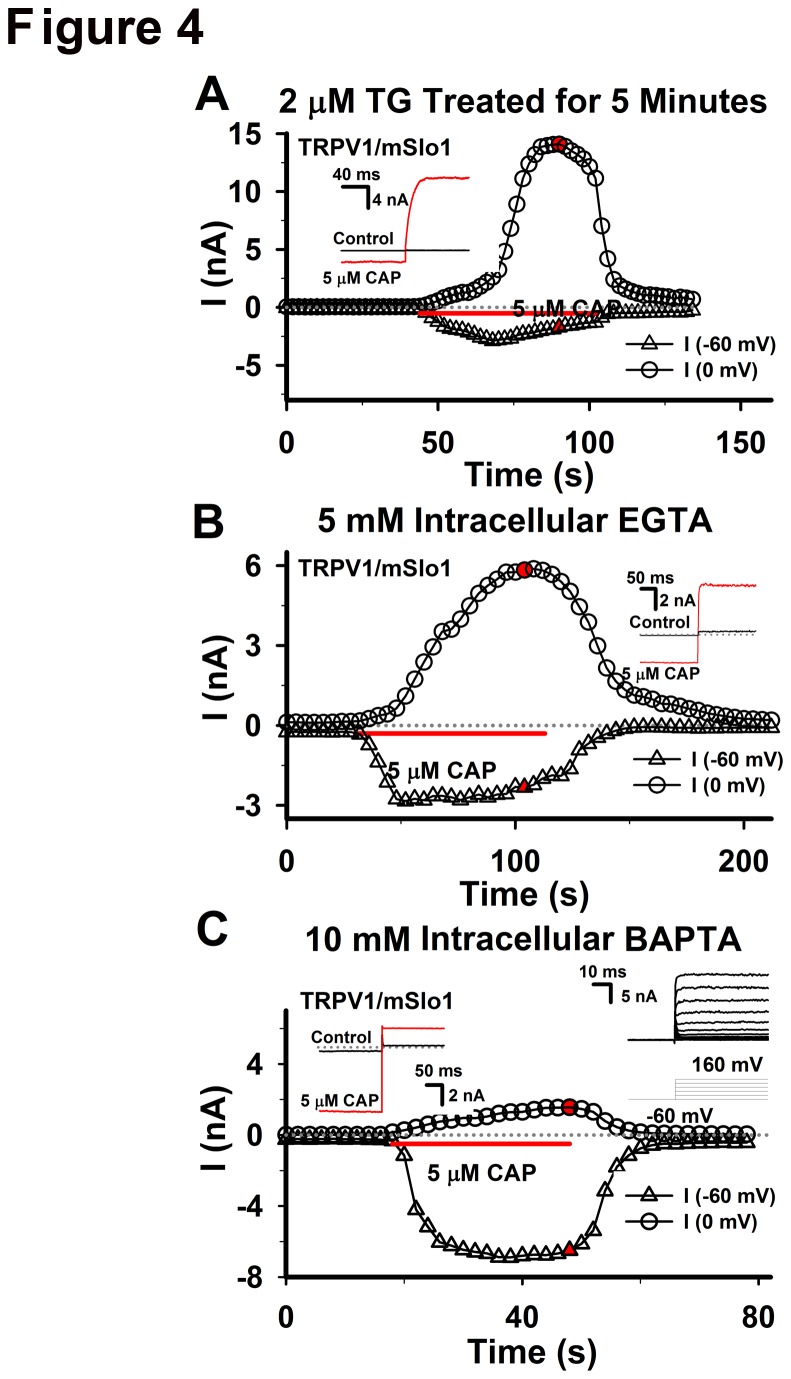
Miscellaneous properties of TRPV1/mSlo1 coupling. A, after treated with 2 μM TG for 5 minutes, the currents of TRPV1/mSlo1, co-expressed in HEK293 cells, and their time courses were obtained as described in Figure 2. The representative traces at -60 and 0 mV are shown in inset as indicated. B, with adding 5 mM EGTA into the intracellular solotion, the currents of TRPV1/mSlo1 and their time courses were obtained as described in Figure 2. The representative traces at -60 and 0 mV are shown in inset as indicated. C, the same as described in B, but for 10 mM BAPTA. The left inset shows the representative traces at -60 and 0 mV and the right inset show the mSlo1 currents activated by a set of voltages ranging from -60 to 160 mV.

Recently, Earley et al. (2011) revealed that TRPV4 formed a signaling complex with Ryanodine Receptors (RyRs) and BK Channels. They demonstrated that TRPV4-dependent Ca^2+^ signals were amplified by opening the RyRs of sarcoplasmic reticulum (SR) to increase Ca^2+^ spark frequency and ultimately activate BK channels. Is it possible that Ca^2+^ is released from intracellular calcium stores or a global concentration in our cases? Therefore, thapsigargin (TG), an inhibitor of SR Ca^2+^ ATPase SERCA pump, was used to empty the intracellular calcium store to examine whether BK channel could be activated again. After treated with 2 μM TG for 5 min, a TRPV1/BK co-transfected HEK293 cell showed strong BK currents with a similar time course as described previously (Figure 4A), suggesting that BK currents were stimulated only by TRPV1 channels. In addition, it was reported that the global Ca^2+^ concentration in the TRPV1-expressed CHO cells, induced by application of various vanilloid agonists, were less than 200 nM [24]. In other words, the global Ca^2+^ is too low to activate BK currents at 0 mV. 

Furthermore, it is important to investigate the sensitivity of TRPV1-BK coupling to Ca^2+^-buffers. With the application of intracellular solutions containing 5 mM EGTA or 10 mM BAPTA for 3-5 minutes, we found that the whole-cell currents of TRPV1-activated BK recorded at 0 mV were unaffected by 5 mM EGTA (Figure 4B), whereas 10 mM BAPTA depressed most of their currents (Figure 4C), indicating that Ca^2+^-sensitive processes are placed within ~20 nm from Ca^2+^ sources (nanodomain) [20,21]. It was reported that 20 mM BAPTA depressed the effect of calcium-dependent inactivation of L-type calcium channel significantly, indicating that BAPTA can chelate the calcium ions at any distance [25]. In contrast, processes with an equal BAPTA/EGTA sensitivity are within 20-200 nm (microdomain). Since 10 mM BAPTA affects significantly BK/TRPV1 currents, but 5 mM EGTA does not, we inferred that BK and TRPV1 were able to form a TRPV1-BK complex. In addition, the previous temperature experiments demonstrated that TRPV1-BK coupling loaded Ca^2+^ to activate BK channels at 0 mV in submilliseconds as Cav-BK coupling did (Figure 2), distances between BK and TRPV1 should be within nanodomains, assuming that one TRPV1 supplies Ca^2+^ ions for one BK [20,26]. According to above facts with their analysis from Cav-BK coupling, TRPV1 should be mainly within ~20 nm of BK.

### TRPV1-BK coupling in DRG cells

**Figure 5:**
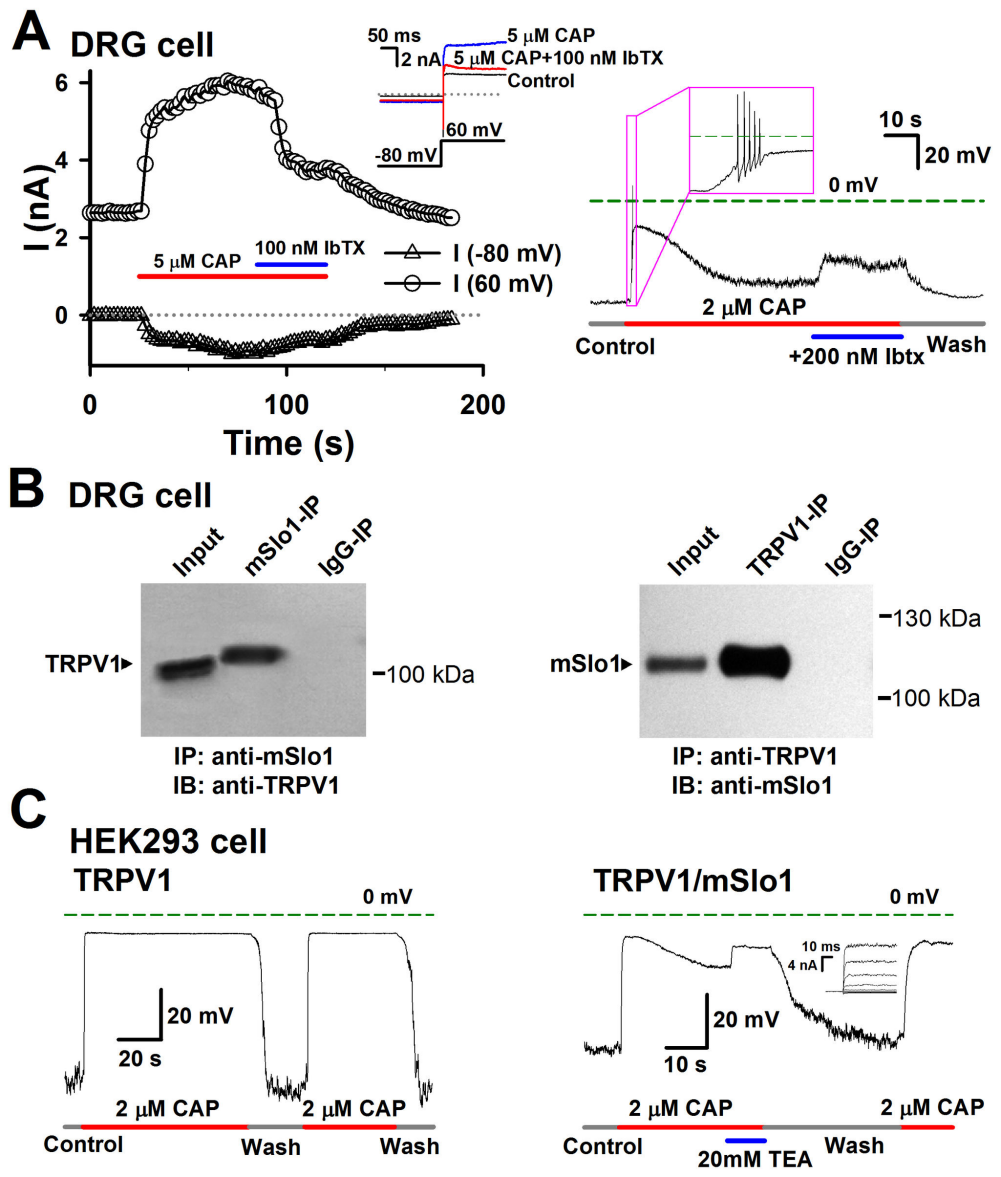
The physiological role of the TRPV1-BK coupling in DRG cells. A, Left, BK currents were activated by an influx of Ca^2+^ through TRPV1 channels in DRG cells. Currents in DRG cells were recorded with repetitive voltage steps from -80 to +60 mV with a time interval of 2 seconds, before, during, and after the application of 5 μM CAP with/without 100 nM Ibtx as indicated by the horizontal bars. The peak currents at -80 and +60 mV are plotted as a function of elapsed time. The representative traces at -80 and +60 mV are shown in inset as indicated. The voltage protocol is placed at the bottom. Right, the action potentials induced by 2 μM CAP and depolarized by 0.2 μM Ibtx in a DRG cell. B, Coimmunoprecipitation experiments carried out on DRG cells. Left: TRPV1 channels can be detected in immunoprecipitates prepared by anti-TRPV1 antibody (mSlo1-IP) (n = 3). Right: mSlo1 channels can be detected in immunoprecipitates prepared by anti-Slo1 antibody (TRPV1-IP) (n = 3). The lane “input” refers to a sample of the original cell extracts from DRG cells. The lane “IgG-IP” respresents a negative control sample, which was immunoprecipitated with homologous rat or goat serum. C, Left, the changes of membrane potentials in the TRPV1 transfected HEK293 cell caused by 2 μM CAP only. Right, the changes of membrane potentials in the TRPV1/mSlo1 co-transfected HEK293 cell caused by 2 μM CAP and 20 mM TEA as indicated. Inset shows the BK currents recorded from an inside-out patch, in the presence of 10 μM Ca^2+^.(n=3-5).

Considering the disadvantages in over-expression HEK293 system such as the non-physiological target protein levels and disrupted intracellular protein environment, we were further interested in examining the TRPV1-BK coupling and its role in native DRG cells. Considering that DRG cell contains multiple K^+^ channels including Kv, BK, SK and so on, we thus used Iberiotoxin (Ibtx), a specific blocker of BK channel, to identify the BK currents activated by Ca^2+^ influx through TRPV1. Before adding capsaicin, there was a Kv current of about 2.7 nA at +60 mV in a DRG cell (Figure 5A left). Here we adopted +60 mV as test voltage in order to minimize calcium influx via calcium channels [27]. With the application of 5 μM capsaicin, the mixture of BK and TRPV1 currents rapidly increased from 2.7 to 5 nA at the early time, then slowly grew, and finally stabilized to a maximal current of 6 nA. After applying the extra 100 nM Ibtx, the current went down to 3.7 nA, indicating that a BK current of ~2.3 nA was activated by Ca^2+^ influx through TRPV1 channels. This BK current from TRPV1-BK coupling may play an important role in depressing the membrane potential during the hyperpolariztion stage of action potentials (APs). Therefore, we inferred that the depolarization process induced by TRPV1 currents would contain a repolarization process in DRG cells due to an augment of the outward BK currents evoked by the Ca^2+^ influxes. As we expected, a DRG cell showed a rapid depolarization with a few of APs and then a slow repolarization by 2 μM Capsaicin (Figure 5A right). After applying with 0.2 μM Ibtx to inhibit BK currents, the cell showed depolarization again. Also, co-immunoprecipitation experiments from native DRG cells were performed to confirm the coupling between TRPV1 and BK. Two highly specific antibodies directed against Slo1 and TRPV1 were used for this target. Immunoblotting with the DRG lysates demonstrated specific Slo1 and TRPV1 bands (Figure 5B, input). Importantly, the Slo1-IP sample demonstrated a strong immunereactive band of TRPV1 subunit (Figure 5B, left), suggesting that Slo1 was able to pull down TRPV1. Furthermore, anti-TRPV1 antibody was able to reciprocally pull down Slo1 (Figure 5B, right). The specificity of the TRPV1-BK coupling was further confirmed by the absence of corresponding band in the control IgG-IP sample. Alternatively, we compared the membrane potentials between the TRPV1- and TRPV1/mSlo1- co-expressed HEK293 cells, because the BK channels also couple with the Cav channels. In Figure 5C left, the TRPV1 expressed cell showed a stable depolarization induced by 2 μM Capsaicin, while the TRPV1/mSlo1 coexpressed cell showed a rapid depolarization following by a slow repolarization clearly induced by an augment of BK currents. This can be further proved by applying with 20 mM TEA (Tetraethylammonium chloride), a BK inhibitor (Figure 5C right). Altogether, this indicates that BK coupling with TRPV1 regulates the membrane potentials in DRG cells.

## Discussion

In this study, major findings are as follows: (1) TRPV1 channel functionally coupled with BK channel independent to the intracellular calcium stores; (2) local Ca^2+^ concentration of BK was up to over 10 μM; (3) immunofluoresence experiments showed that BK and TRPV1 were colocalized mainly on the plasma membrane; (4) coimmunoprecipitation experiments showed that BK associated with TRPV1 to form a TRPV1-BK complex; (5) TRPV1-BK coupling exists in DRG cells. Altogether, our results revealed that a tightly TRPV1-BK coupling, of which form a TRPV1-BK complex, could serve to resist the pains induced by various vanilloid agonists, acid and temperature in peripheral nervous system, which may also explain the adaptability of animals to special environment, such as the extremely higher (>42 °C) temperature. Certainly, this adaptability comes from both the desensitization of TRPV1 channel and the negative feedback of BK channel. 

**Figure 6:**
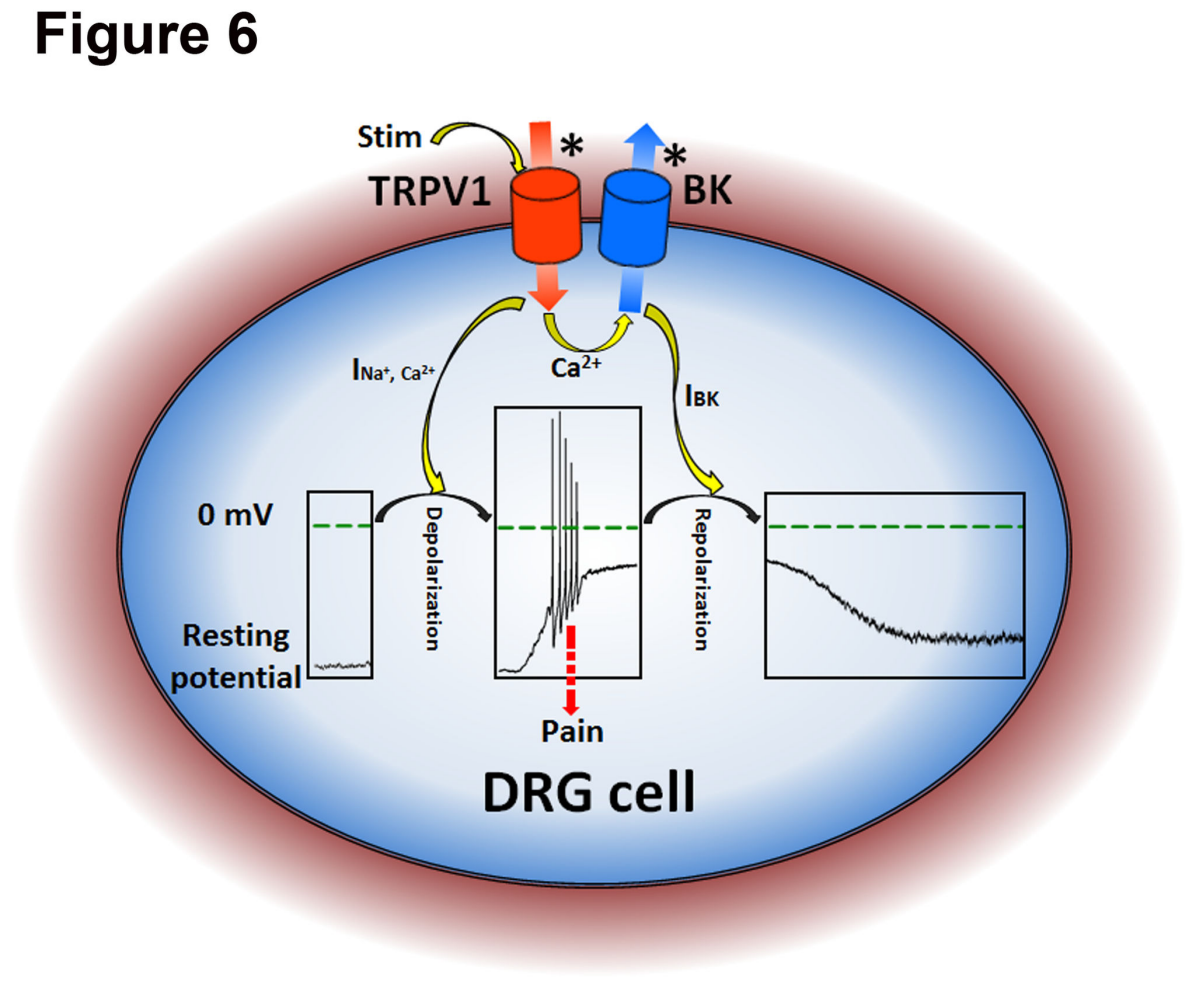
A schematic diagram for illustrating the pain-transduction pathway in DRG cell. A star * denotes a channel activated. Stim means stimuli for TRPV1 such as capsaicin, temperature and H^+^. The inner boxes show changes on the membrane potentials in DRG cells. The I_Na_^+^_,Ca_^2+^ and I_BK_ are the currents of TRPV1 and BK channels, respectively.

TRPV1 channels are mainly expressed in sensory neurons such as DRG cells, where BK channels are also present [11,17]. Moreover, TRPV1 is highly permeable to Ca^2+^, while BK channels can be activated by intracellular Ca^2+^. Thus both their expression patterns and functions suggest that the two are functionally coupled, where the opening of TRPV1 leads to elevate intracellular Ca^2+^, in turn leading to activation of BK channels. Since BK has the largest single channel conductance making it an idea candidate for maintaining the resting potentials of cells, the activation of BK by Ca^2+^ influx induced by TRPV1 channels will repolarize the membrane potentials as a negative feedback to the depolarization of nociceptors evoked by TRPV1 to decrease the strengthen of stimulation as self-protection (Figure 6). Also we should notice that not all the DRG cells had inward current by applying 5 μM capsaicin, and there was no significant change in outward current (data not shown). This meant in some cells, the TRPV1 channels might be degraded or destroyed by enzymes during preparation, BK channel could not be activated by the absence of calcium influx. This was the same case as a TRPV1 channel knockdown cell.

It is really plausible to say that all the BK and TRPV1 can form channel-channel complexes as we have to consider many uncertain things, such as, multiple TRPV1 vs a BK, uniform or non-uniform distribution of channels, direct or indirect (via the third protein) association, fraction of physical association and so on. In the example of DRG (Figure 5A), maximal TRPV1 current at -80 mV was only 1 nA, which means the number of TRPV1 n(TRPV1)=125, assuming that the single-channel conductance of TRPV1 g(TRPV1) =100 pS and the reversal potential of TRPV1 V_reversal_ (TRPV1)= 0 mV. Similarly, the BK current of 3.7 nA at 60 mV confers n(BK)=155, assuming that g(BK) = 200 pS and V_reversal_ (BK)=-60 mV. Assuming that the diameter of a spheral cell is about 20 μm, this implies that the average distance between TRPV1 and BK is larger than 2 μm. Therefore, their distribution on plasma membrane should not be a uniform distribution. In other words, the global (or average) Ca^2+^ concentration is incapable to activate BK. Alternatively, we found that both the confocal images of mSlo1-TagGFP (left panel) and TRPV1-myc (middle panel) showed clearly green and red edges in cluster (Figure 3A). This suggests that channels distribute respectively into clusters on the plasma membrane. We deduce that clusters are derived from those trafficking vesicles of BK and TRPV1. Vesicles should be in clusters if they were transported to the surface of membrane via the same “railways”, e.g. microtubulin and actin. In this case, BK and TRPV1 channels should be closed enough to associate together directly or indirectly via the third protein. All the hypotheses should be further investigated in the future. 

Both BK and TRPV1 are abundantly expressed in almost all types of DRG neurons. The vanilloid receptor TRPV1 in DRG neurons senses various outside signals including acids, pungent agents and heat. The BK current derived from the TRPV1-BK coupling may play a critical role in enhancing hyperpolarization to prevent noxious pain induced by vanilloids, acids and heat.
